# Pharmacogenetics association with long-term clinical evolution in a kidney transplant patients cohort

**DOI:** 10.1016/j.crphar.2025.100230

**Published:** 2025-07-24

**Authors:** Luis Sendra, Gladys G. Olivera-Pasquini, Enrique G. Zucchet, Fabiana D.V. Genvigir, María Isabel Beneyto, Julio Hernández-Jaras, María José Herrero, Salvador F. Aliño

**Affiliations:** a Gene Therapy and Pharmacogenomics, Department of Pharmacology, University of Valencia, 46010, Valencia, Spain; b Pharmacogenetics and Gene Therapy Unit, La Fe Health Research Institute, 46026, Valencia, Spain; c Department of Clinical and Toxicological Analyses, School of Pharmaceutical Sciences, University of Sao Paulo, 05508-000, Sao Paulo, Brazil; d Urology and Nephrology Service, University and Polytechnic La Fe Hospital, 46026, Valencia, Spain

**Keywords:** Transplantation, Immunosuppression, Pharmacogenetics, Long-term evolution, Gene variants, Clinical evolution

## Abstract

**Background:**

Pharmacogenetic variability has been reported to influence the efficacy and safety of immunosuppressive therapies in early stages of kidney transplantation. This study investigates long-term associations between pharmacogene variants and clinical outcomes in a cohort of kidney transplant recipients over a 12-year follow-up.

**Materials and methods:**

We analyzed 37 SNPs from 14 genes related to drug metabolism and transport in 79 kidney transplant patients. Clinical parameters, including survival, renal function, tumor occurrence, and pharmacokinetics of tacrolimus, were evaluated. Logistic regression and Kaplan-Meier analyses assessed associations between gene variants and clinical outcomes.

**Results:**

Variants in metabolizer (CYP3A5, CYP2B6) and transporter genes (ABCB1, ABCC2) were associated with 12-year survival. Increased tumor risk correlated with ABCC2 variants in donors and decreased risk with CYP2B6 rs3745274 in recipients. Renal function was influenced by variants in ABCB1, ABCC2, CYP3A5, CYP3A4, and CYP2B6. Tacrolimus dose-dependent concentration was affected by variants in CYP3A4, CYP3A5, CYP2C19, ABCB1, and SLCO1B1. Increased nephrotoxicity risk was associated with CYP2C19 rs4244285 and reduced by SLCO1B1 rs2306283 AA and AG variants. Gene variant interactions between metabolizer and transporter genes were also associated with altered risk of events incidence.

**Discussion:**

Our findings support that pharmacogene variants influence transplant outcomes. Notable associations include survival related to ABCB1 and ABCC2 variants, tumor occurrence linked to CYP2B6 rs3745274, and renal function affected by multiple pharmacogenes. Variants in CYP2C19 and SLCO1B1 significantly impacted tacrolimus pharmacokinetics and nephrotoxicity risk. These results underline the importance of pharmacogenetic testing for personalized management in kidney transplantation, although further validation in larger cohorts is necessary.

## Highlights

• CYP2B6 rs3745274 GT variant in recipients was associated to lower tumor and DM occurrence risk.
• CYP2C19∗2 genotype associated with high dose-adjusted concentrations (C0/D) of tacrolimus and higher risk of suffering nephrotoxicity.
• The ABCB1 rs2235013 GA variant in donors proved associated to reduced risk of chronic rejection occurrence.
• The presence of some CYP3A5–SLCO1B1 and CYP3A5–ABCB1 variants interaction increased the risk of patients' exitus during the follow-up.
• The interaction of some CYP2B6–ABCB1 variants increased the risk of nephrotoxicity and acute rejection occurrence.

## Graphical abstract

**Figure:**
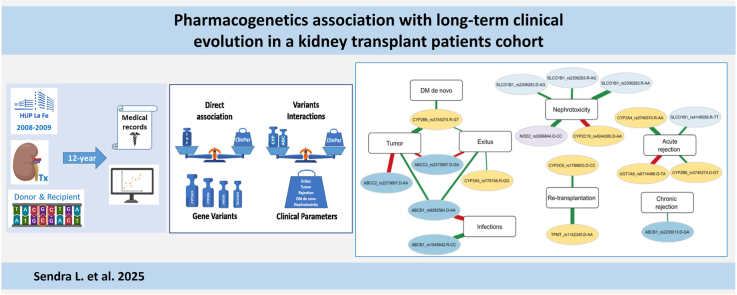

## 1 Introduction

Kidney transplantation is currently the only curative therapeutic option for end-stage renal diseases that severely impair normal kidney function. Notably, the kidney is the most frequently transplanted solid organ worldwide, accounting for approximately 65 % of all transplants. This intervention necessitates lifelong administration of multiple pharmacological agents aimed at modulating the patient's immune response to promote graft tolerance while maintaining defenses against infectious agents. The therapeutic regimen typically includes corticosteroids (initially and in cases of suspected rejection) and chronic immunosuppressive therapy, particularly with calcineurin inhibitors, among which tacrolimus is the most commonly prescribed. All patients in our cohort received tacrolimus, as it significantly reduces acute rejection rates and improves long-term graft survival (Mayer et al., 1997).

Despite its effectiveness, tacrolimus has a narrow therapeutic range and is associated with several adverse effects, including nephrotoxicity, increased susceptibility to infections, post-transplant diabetes mellitus, neurotoxicity, hypertension, and malignancies, which may limit its use (Ekberg et al., 2007). Furthermore, significant interindividual variability in plasma concentrations is frequently observed, both over time within the same patient and across different patients. This variability can be attributed to various factors, including concomitant drug use, dietary habits, herbal supplements, and intrinsic factors such as sex, age, serum albumin levels, hematocrit, and genetic variants. Of particular relevance are genetic polymorphisms in pharmacogenes encoding proteins involved in the transport and metabolism of tacrolimus, as these can affect plasma drug levels, potentially leading to reduced therapeutic efficacy and/or increased incidence of adverse effects.

Tacrolimus pharmacokinetics involve several enzymes, including P-glycoprotein (encoded by the ABCB1 gene), an ATP-dependent efflux pump that facilitates the excretion of tacrolimus from intestinal cells into the lumen, promoting its elimination via feces or metabolism. Tacrolimus undergoes extensive metabolism primarily in the liver and intestinal wall by CYP3A5 and CYP3A4 enzymes, generating at least eight distinct metabolites (Iwasaki, 2007), some of which may contribute to tacrolimus toxicity (de Jonge et al., 2009). These metabolites are excreted predominantly via bile (∼97 %) and, to a lesser extent, urine (∼2 %). Despite the well-established involvement of these enzymes, data on their specific roles in tacrolimus metabolism remain inconsistent, and the pharmacological activity of tacrolimus metabolites requires further investigation. Less than 1 % of tacrolimus is excreted unchanged in urine and feces (Henkel et al., 2023). Thus, genetic variants in the genes encoding these proteins (pharmacogenes) that affect their function could alter drug transport and metabolism, potentially leading to plasma tacrolimus levels that fall outside the therapeutic range, thereby reducing immunosuppressive efficacy and increasing the risk of adverse events, particularly in long-term therapeutic regimens. Consequently, pharmacogenetic studies could help predict patient outcomes in certain cases. Although short-term studies on kidney transplantation have been previously reported, the information of longer follow-up periods is scarce. As far as we know, no previous study covers the period of time we consider in the present work.

Most previous studies investigating potential pharmacogenetic variants associated with treatment success have focused on single or small sets of SNPs and short follow-up periods. Given the incomplete understanding of the mechanisms involved in drug metabolism, there is growing evidence that simultaneous analysis of broader panels of variants, including those in additional pharmacogenes, could help identify new associations and rule out nonspecific ones (Steinbach et al., 2022; Niedrig et al., 2021). Therefore, in this study, we evaluated a panel of 37 SNPs previously reported as relevant variants located in 14 pharmacogenes involved in key ADME (Absorption, Distribution, Metabolism, and Excretion) mechanisms. Considering the unique context of organ transplantation, both donors and recipients were genotyped. This study was conducted in a cohort of 79 kidney transplant recipients who underwent transplantation in 2008 and 2009 at Hospital Universitario y Politécnico La Fe (Valencia, Spain). Clinical events commonly observed in transplant patients were collected from medical records during a 12-year follow-up period, and potential associations with pharmacogene variants were analyzed.

## 2 Material and methods

### 2.1 Patients

This study was conducted in accordance with the Declaration of Helsinki and received approval from the Institutional Ethics Board Committee of Hospital Universitari i Politècnic La Fe, Valencia, Spain (registry number: 2008/0263). Written informed consent was obtained from all participants. The study included adult kidney transplant recipients (aged >18 years) who underwent transplantation between March 2008 and December 2009 and were treated with tacrolimus (n = 79). Three patients were excluded due to early loss of clinical follow-up or lack of genetic data. Demographic, clinical, and laboratory data were retrospectively obtained from electronic health records with the approval of the Hospital Ethics Committee of Medicines Research (reference: 2021-370-1).

### 2.2 Immunosuppressive therapy

Tacrolimus (Prograf®, Astellas Pharma Inc., Chuo-Ku, Tokyo, Japan) was administered orally within 24 h post-surgery, unless clinical complications arose. The initial dose was 0.1 mg/kg/day, divided into two doses daily. Dosage adjustments were made to achieve target trough levels of 10–15 ng/mL during the first 3 months, 5–15 ng/mL up to 12 months, and 5–10 ng/mL thereafter. Immunosuppressant therapy protocol included also mycophenolate (2 g/day), which was withdrawn when possible, and corticoids. Methylprednisolone (10 mg/kg) was administered intravenously during surgery, with the dosage gradually reduced and transitioned to oral prednisolone one week post-transplant. Prednisolone dosage was then tapered and discontinued when clinically feasible.

### 2.3 Tacrolimus measurements

Tacrolimus blood concentrations were measured using the ACMIA immunoassay method (Dimension; Siemens Healthineers, Erlangen, Germany) from whole-blood samples collected 24 h after tacrolimus administration (C_0_, ng/mL) at various follow-up points: 7 and 14 days; 1, 2, 3, 6, 9, and 12 months; and 3, 6, 9, and 36 months. Dose-adjusted trough concentrations (C/D ratio) were calculated by dividing the tacrolimus trough concentration (C_0_) by the corresponding 24-h dose on a mg/kg basis (ng/mL/mg/kg) at each time point.

### 2.4 Single nucleotide polymorphism identification

Genotyping of single nucleotide polymorphisms (SNPs) was performed using the MassARRAY platform (SEQUENOM, now Agena Bioscience, San Diego, CA, USA) at the Central Service of Experimental Research Support (SCSIE), Faculty of Medicine, Universitat de València, Spain. Briefly, genomic DNA was extracted from whole blood collected in EDTA tubes from transplant recipients and donors. DNA was purified from 200 μL of blood using the Ultra Clean Blood Spin DNA Isolation Kit (MoBio Laboratories Inc., Carlsbad, CA, USA) following the manufacturer's instructions. DNA concentration and purity were assessed using a NanoDrop spectrophotometer (NanoDrop Technologies Inc., Wilmington, DE, USA), and the DNA was stored at −20 °C until use. All samples were genotyped in triplicate to ensure the quality of the technique.

### 2.5 SNP panel

The SNPs evaluated (Table S1) were selected based on previous studies associating genes with clinical outcomes in solid organ transplant recipients, along with additional polymorphisms in genes encoding drug transporters and other signaling pathways of potential interest.

### 2.6 Clinical outcomes

Survival rate was assessed by recording death from any cause during the follow-up period (exitus). Tumor incidence included only de novo tumors that occurred during follow-up. Diabetes mellitus and arterial hypertension were diagnosed following standard clinical procedures. Severe infections in transplanted patients were classified as de novo infections. Nephrotoxicity was defined as an increase in serum creatinine levels greater than 0.5 mg/dL or 20–25 % above pre-transplant baseline, coinciding with elevated tacrolimus levels that normalized upon tacrolimus dose reduction. Other causes of renal impairment, such as fever, infection, graft swelling, oliguria, bleeding, dehydration, increased resistive index on Doppler ultrasonography, or ureteral obstruction, were excluded. Renal clearance was calculated using the CKD-EPI (Chronic Kidney Disease Epidemiology Collaboration) estimated glomerular filtration rate, which accounts for serum creatinine, age, sex, and ethnicity. The CKD-EPI equation, expressed as a single equation, is GFR = 141 × min(Scr/κ, 1)^α^ × max(Scr/κ, 1)^−1.209^ × 0.993^Age^ × 1.018 [if female] x 1.159 [if black], where Scr is serum creatinine, κ is 0.7 for females and 0.9 for males, α is −0.329 for females and −0.411 for males, min indicates the minimum of Scr/κ or 1, and max indicates the maximum of Scr/κ or 1. Chronic cellular kidney rejection was defined according to the Banff consensus, which requires the presence of inflammation in areas of interstitial fibrosis and tubulitis, necessitating biopsy for diagnosis.

### 2.7 Statistical analyses

Statistical analyses were conducted using SPSS software (version 28; IBM, NY, USA). Multivariate logistic regression was used to evaluate the association of genetic variants with the risk of death (exitus), tumor occurrence, acute and chronic rejection, nephrotoxicity, re-transplantation, diabetes mellitus, arterial hypertension, and infections during follow-up. All gene variants were included as variables, with age and gender as covariates. Hazard ratios (HR) and 95 % confidence intervals (CI) were calculated for clinical outcomes associated with each variant. Survival and tumor incidence were analyzed using logistic regression with stepwise selection and false discovery rate correction in SPSS. The interaction between significant variants located in metabolic and transport pharmacogenes were also studied by logistic regression employing SPSS to determine whether the combination of variants in pairs of genes could alter the risk of clinical events incidence in patients. The time evolution of survival during follow-up, stratified by significant genetic variants, was represented by Kaplan–Meier curves and analyzed using the log-rank test. Renal clearance (CKD-EPI, mL/min/1.73 m^2^) and tacrolimus trough concentrations, adjusted for dose (C/D, ng/mL/mg/kg), were recorded for each patient over the 12-year follow-up. For SNPs showing significant associations with clinical parameters, renal clearance and tacrolimus concentration curves were plotted using Prism v.07 (GraphPad Software®, Boston, MA, USA). Comparisons between patients with different SNP genotypes were made using t-tests for two-allele variants and one-way ANOVA for variants with three genotypes. A p-value <0.05 was considered statistically significant in all analyses. Individual gene variants-clinical parameters associations were represented with Cytoscape software (version 3.10.3; Cytoscape Consortium)

## 3 Results

### 3.1 Pharmacogene variants association with survival

The potential association between exitus during the follow-up period, regardless of the precise moment, and any of the pharmacogene variants determined was evaluated by multivariate logistic regression, including all variants in the analysis. Different variants in transporter and metabolism genes showed a significant association with the risk of exitus, with the latter being excluded after applying the false discovery rate penalization (Table 1). The AA variant in the rs9282564 SNP of the donor's ABCB1 gene was significantly associated (p-value: 0.029) with a reduced risk (OR: 0.116) of exitus during the follow-up period. Conversely, patients receiving a graft from a donor with the GA genotype in rs2273697 of ABCC2 were significantly associated (p-value: 0.021) with an increased risk (OR: 4.133) of exitus.

**Table 1:** Pharmacogene variants associated with probability of survival during the follow-up.

Exitus	Variant	D/R	Significance		OR	OR CI (95 %)	
Gene_SNP							
			Log Regression	Post FDR		Inferior	Superior
ABCB1_rs9282564	AA	D	0.021	**0.029**	0.116	0.019	0.726
ABCC2_rs2273697	GA	D	0.016	**0.021**	4.133	1.301	13.125
CYP2B6_rs3745274	GT	R	0.044	0.060	0.224	0.052	0.960
CYP3A5_rs776746	GG	R	0.048	0.066	0.264	0.071	0.989

SNP: Single Nucleotide Polymorphism; D: donor; R: recipient; FDR: false discovery rate.

OR: Odds Ratio; CI: confidence interval.

Bold numbers in significance are those lower than 0.05, which is considered the statistical significance threshold.

**Fig. 1:**
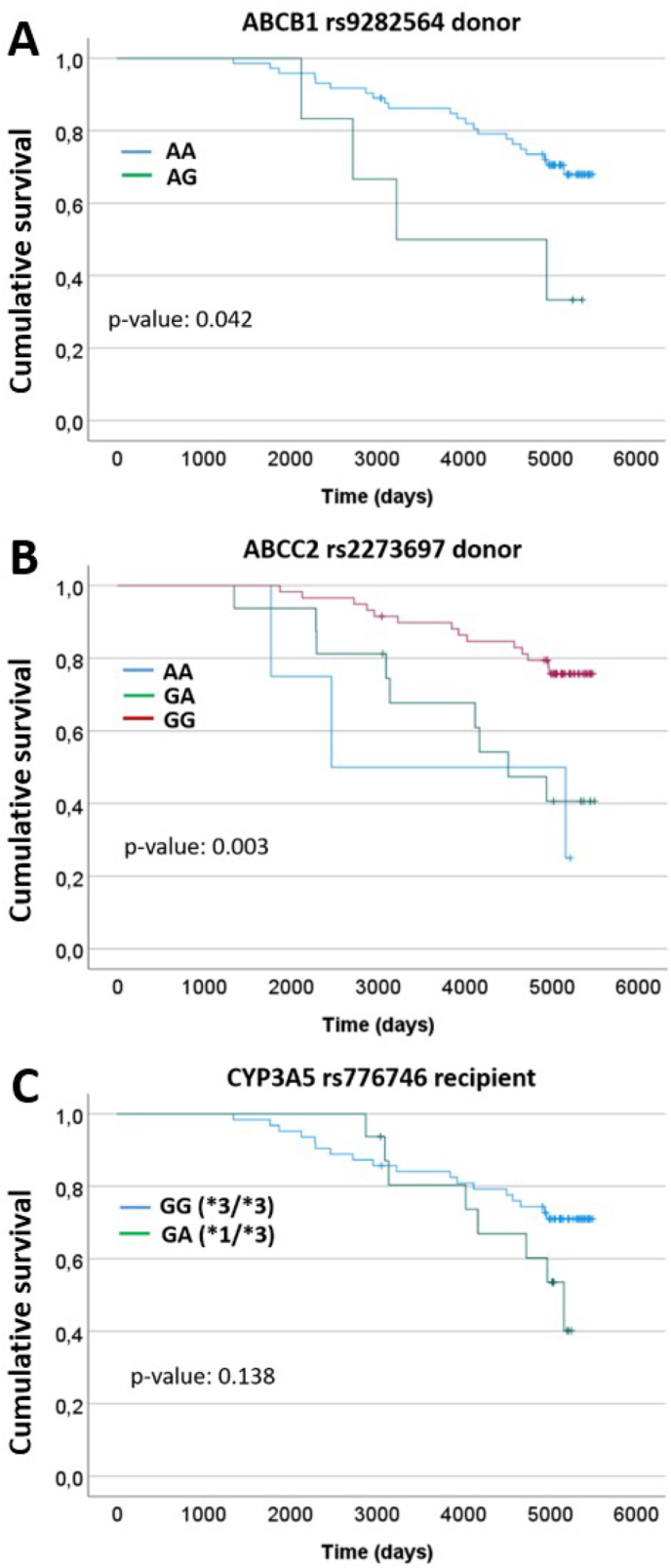
Kaplan-Meier survival curves with significant differences according to the pharmacogene variants in SNPs associated with exitus. CYP3A5 was also included because of the relevance of the gene in tacrolimus metabolism; although not significant, the GG (∗3/∗3) and GA (∗1/∗3) curves present different behavior. Statistical test: log-rank.

The number of patients presenting each genotype of the SNPs associated with exitus and the occurrence of this event is represented in Table S2 (supplementary material). To confirm the effect of the associated gene variants on patient survival during the 12-year follow-up, Kaplan-Meier curves were plotted. The AG variant in ABCB1 gene rs9282564 of the donor was significantly associated (p-value: 0.042; log-rank) with reduced cumulative survival compared to the AA variant (Fig. 1A). It was observed that the genotype in the donor's ABCC2 rs2273697 could significantly (p-value: 0.003; log-rank) affect survival (Fig. 1B), with the GG variant providing a higher rate during the entire follow-up compared to AA and GA. Although the CYP3A5 rs776746 did not show a significant association with survival in logistic regression, the Kaplan-Meier curve was also represented (Fig. 1C) due to its special relevance and clinical evidence on the response to tacrolimus. No significant difference was observed by log-rank either, but it should not be discarded that the GG variant (∗3/∗3) could offer an advantage, especially at late post-transplantation stages.

### 3.2 Pharmacogene variants association with tumor incidence

The association between pharmacogene variants and cancer occurrence, regardless of the exact moment during the 12-year follow-up, was also evaluated (Table 2), given the influence that imbalanced immunosuppression could present on tumor control. Multivariate logistic regression using all pharmacogene variants showed that recipients carrying the GT heterozygous genotype in CYP2B6 rs3745275 could present an advantage (OR: 0.099; p-value: 0.048). Conversely, a significant (p-value: 0.043) association with an increased risk (OR: 13.09) of tumor appearance during the follow-up was found in patients receiving a kidney with the AA variant in ABCC2 rs2273697.

**Table 2:** Pharmacogene variants associated with probability of tumor occurrence during the follow-up.

Tumor	Variant	D/R	Significance		OR	OR CI (95 %)	
Gene_SNP							
			Log Regression	Post FDR		Inferior	Superior
CYP2B6_rs3745274	GT	R	0.035	**0.048**	0.099	0.012	0.850
ABCC2_rs2273697	AA	D	0.032	**0.043**	13.091	1.241	138.105
	GA	D	0.043	0.058	3.394	1.037	11.103
ABCB1_rs9282564	AA	D	0.042	0.057	0.120	0.015	0.930

SNP: Single Nucleotide Polymorphism; D: donor; R: recipient; FDR: false discovery rate.

OR: Odds Ratio; CI: confidence interval.

Differences in population distribution between the presence and absence of cancer during the follow-up, depending on the specific genotype in these SNPs, were also observed (Table S3; supplementary material).

### 3.3 Pharmacogene variants association with clinical parameters affecting renal function

To identify associations between pharmacogenes and other clinical parameters affecting kidney function that could arise due to the variable response to administered drugs, multivariate logistic regression was also performed, considering all pharmacogene variants as independent variables and chronic and acute rejection, nephrotoxicity, and re-transplantation as dependent variables. Results are shown in Table 3, and it can be observed that the GA variant in ABCB1 rs2235013 is associated with a reduced risk of chronic rejection (p-value: 0.016; OR: 0.13). Regarding acute rejection, statistical analysis showed that the CYP3A4 rs2740574 AA variant in the recipient could offer a protective effect (OR: 0.080; p-value: 0.047) compared to other variants at that location. The presence of the TT variant in SLCO1B1 rs4149056 in the recipient and the GT variant in CYP2B6 rs3745274 in the donor were also statistically associated with a reduced risk (OR: 0.16 and 0.2, respectively) of acute rejection. On the contrary, the TA variant in UGT1A9 in rs6714486 of donor showed an association with an increased risk of acute rejection, although the imbalanced distribution of patient genotypes prevents us from considering such a high odds ratio.

**Table 3:** Pharmacogene variants associated with the probability of clinical events occurrence regarding renal function during the follow-up.

	Gene_SNP	Variant	D/R	Significance		OR	OR CI (95 %)	
				Log Reg	Post FDR		Inferior	Superior
**Chronic rejection**	ABCB1_rs2235013	GA	D	0.012	**0.016**	0.133	0.028	0.643
**Acute rejection**	CYP3A4_rs2740574	AA	R	0.035	**0.047**	0.080	0.008	0.833
	SLCO1B1_rs4149056	TT	R	0.018	**0.024**	0.157	0.034	0.725
	CYP2B6_rs3745274	GT	D	0.020	**0.027**	0.199	0.051	0.777
	UGT1A9_rs6714486	TA	D	0.015	**0.020**	32.116	1.952	528.327
**Nephrotoxicity**	SLCO1B1_rs2306283	AA	R	0.007	**0.009**	0.076	0.012	0.492
		AG	R	0.038	0.051	0.227	0.056	0.919
	NOD2_rs2066844	CC	D	0.026	**0.035**	0.015	0.000	0.598
	SLCO1B1_rs2306283	AG	D	0.018	**0.024**	0.108	0.017	0.679
	CYP2C19_rs4244285	AA	D	0.019	**0.026**	11.652	1.509	89.957
**Re-transplantation**	TPMT_rs1142345	AA	D	0.015	**0.020**	0.048	0.004	0.559
	CYP2C9_rs1799853	CC	D	0.031	**0.042**	0.102	0.013	0.810

SNP: Single Nucleotide Polymorphism; D: donor; R: recipient; FDR: false discovery rate; OR: Odds Ratio.

CI: confidence interval.

When considering nephrotoxicity, results indicate that the AA and AG variants in SLCO1B1 in the recipient, and the AA variant in this SNP in the donor, were associated with a reduced risk of nephrotoxicity (OR: 0.08, 0.23, and 0.11, respectively). The CC variant in donor NOD2 rs2066844 could also reduce that risk (OR: 0.015). An increased risk of nephrotoxicity was observed in patients receiving grafts carrying the AA variant in CYP2C19 rs4244285. Lastly, a reduced risk of re-transplantation was observed in patients whose implanted kidneys carried the AA variant in TPMT rs1142345 and the CC variant in CYP2C9 rs1799853 (OR: 0.048, p-value: 0.02; and OR: 0.10, p-value: 0.042, respectively). Genotype distribution in patients presenting the different clinical events involved in renal malfunction studied is shown in Table S4 of supplementary material.

### 3.4 Pharmacogene variants association with other clinical parameters with high Prevalence in transplanted patients

**Fig. 2:**
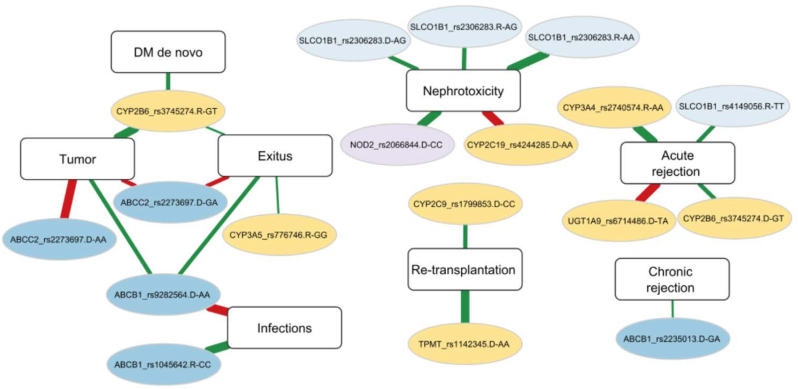
Scheme of significant associations between pharmacogenes variants and the clinical parameters studied. R: Recipient; D: Donor. Yellow nodes: variants in metabolizer genes; Blue nodes: variants in transporter genes. Green lines: reduced risk; Red lines: increased risk; Red thin line: OR 2.0–5.0; Red medium line: OR 5.0–10.0; Thick red line: OR > 10.0. Green thin line: 0.5–0.2; Green medium line: OR 0.2–0.1; Green thick line: OR < 0.1. (For interpretation of the references to colour in this figure legend, the reader is referred to the Web version of this article.)

Post-transplant diabetes mellitus is a common occurrence in kidney transplant patients and can be associated with greater mortality, graft failure, and increased risk of infections, also favored by drug-induced immunosuppression. The potential association of higher risk of these clinical side effects with pharmacogene variants was also evaluated (Table 4). Genotype distribution in patients presenting diabetes mellitus and infections is shown in Table S5 (supplementary material). The scheme in Fig. 2 summarizes the pharmacogene variants-clinical parameters associations obtained in the study. This highlights the relevance of different variants that can alter one or more body functions.

**Table 4:** Pharmacogene variants associated with occurrence risk of diabetes mellitus and infections during the follow-up.

Clinical parameter	Gene_SNP	Variant	D/R	Significance		OR	OR CI (95 %)	
				Log Regression	Post FDR		Inferior	Superior
**DM de novo**	CYP2B6_rs3745274	GT	R	0.056	0.056	0.273	0.072	1.033
**Infections**	ABCB1_rs1045642	CC	R	0.002	**0.003**	0.087	0.019	0.393
	ABCB1_rs9282564	AA	D	0.004	**0.005**	23.800	2.776	204.039

SNP: Single Nucleotide Polymorphism; D: donor; R: recipient; FDR: false discovery rate; OR: Odds Ratio.

CI: confidence interval.

### 3.5 Effect of pharmacogene variants on tacrolimus pharmacokinetics associated with any clinical parameters studied

**Fig. 3:**
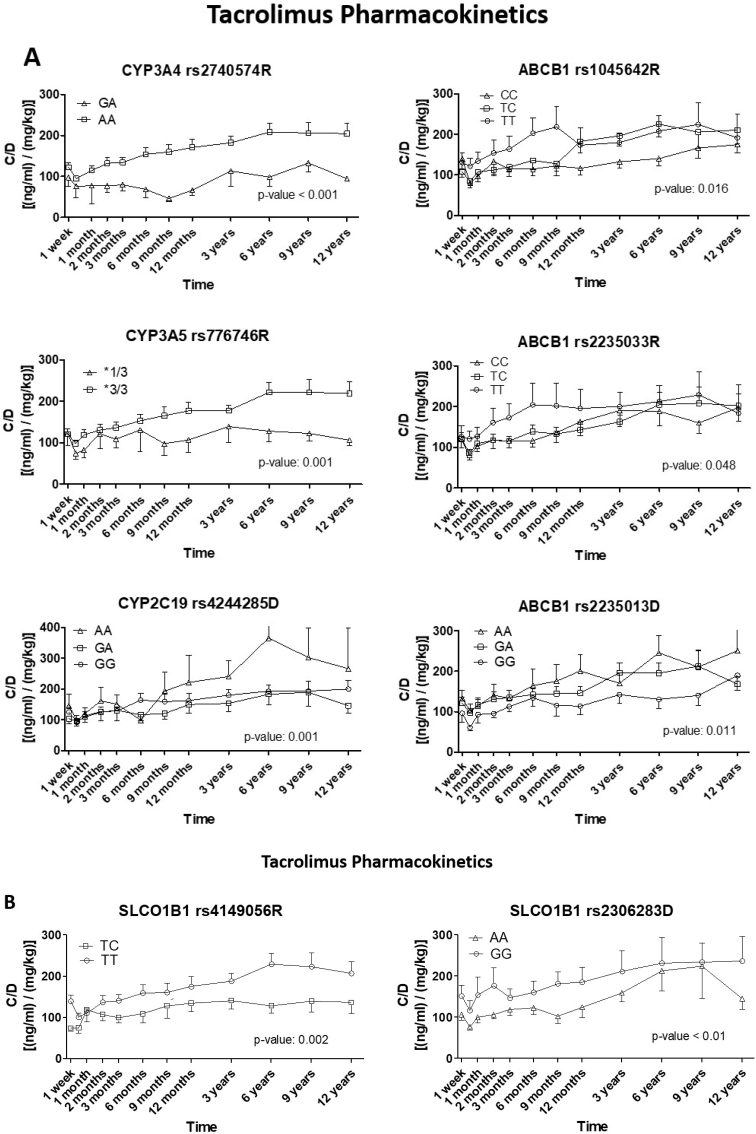
Dose-adjusted tacrolimus concentration [(ng/ml)/(mg/kg)] during the follow-up grouped by pharmacogene variants. Statistical test: *t*-test when there were 2 possible variants; one-way ANOVA for 3 or more.

Dose-adjusted tacrolimus concentration (C/D) was measured in each patient at different time points during the entire follow-up, and the results were compared depending on the variants of each of the SNPs associated with clinical parameters (Fig. 3). Significantly different concentrations depending on metabolism and active transport (ABCB1) pharmacogenes are presented in Fig. 3A (left and right panels, respectively). Results show that patients with the AA variant in CYP3A4 rs2740574 (top left graph) have significantly (p-value <0.001) higher dose-adjusted tacrolimus plasma levels (C/D ratio) during the entire follow-up, with a tendency to increase over time, whereas GA variant patients present sustained lower steady levels. This difference reaches up to 2-fold, 200 vs. 100 [(ng/ml)/(mg/kg)] from the 6th year onwards. CYP3A5 is the main enzyme involved in tacrolimus metabolism, and variants have been described to affect the plasma levels of this drug. The ∗3 haplotype, defined by the presence of G instead of A at rs776746, plays an important role, and pharmacogenetics-driven dose adjustment has been established. In this sense, homozygous patients for this variant (∗3/∗3) presented increasing C/D ratios of tacrolimus during the follow-up, reaching levels that exceed 200 [(ng/ml)/(mg/kg)], which was significantly (p-value <0.001) higher than in heterozygous (∗1/∗3) patients, whose levels remained constant around 100 [(ng/ml)/(mg/kg)]. In the case of CYP2C19, it can be observed that the GG and GA variants in donors’ rs4244285 exhibited similar behavior, whereas patients receiving a kidney graft with the AA variant showed higher levels of tacrolimus, especially from 3 years onwards, achieving high C/D values over 300 [(ng/ml)/(mg/kg)] that would suggest the need of dose reduction.

Regarding active transport pharmacogenes (right panels), only variants in the ABCB1 gene proved significant. Patients with the TT variant in both rs1045642 and rs2235033 SNPs presented significantly (p-value: 0.005 in both cases) higher ratios of tacrolimus C/D than those with any of the other genotype combinations. Patients receiving a kidney graft from a donor with the GG variant in rs2235013 had lower (p-value: 0.011), more sustained dose-adjusted concentrations of the drug, slightly over 100 [(ng/ml)/(mg/kg)]. On the other hand, our results showed (Fig. 3B) significant differences between variants present in genes encoding passive transport proteins, such as SLCO1B1. In this regard, patients with the TT variant in rs4149056 nearly doubled the dose-adjusted tacrolimus concentration (C/D) observed with TC (p-value: 0.002), with the greatest differences observed from 3 years onwards. When comparing tacrolimus C/D in patients receiving kidney grafts from donors with the AA or GG variant in rs2306283, significant differences (p-value: 0.01) were observed during the early post-transplantation phase, which tended to reduce over time.

### 3.6 Effect of pharmacogene variants associated with any clinical parameters studied on renal function (clearance)

**Fig. 4:**
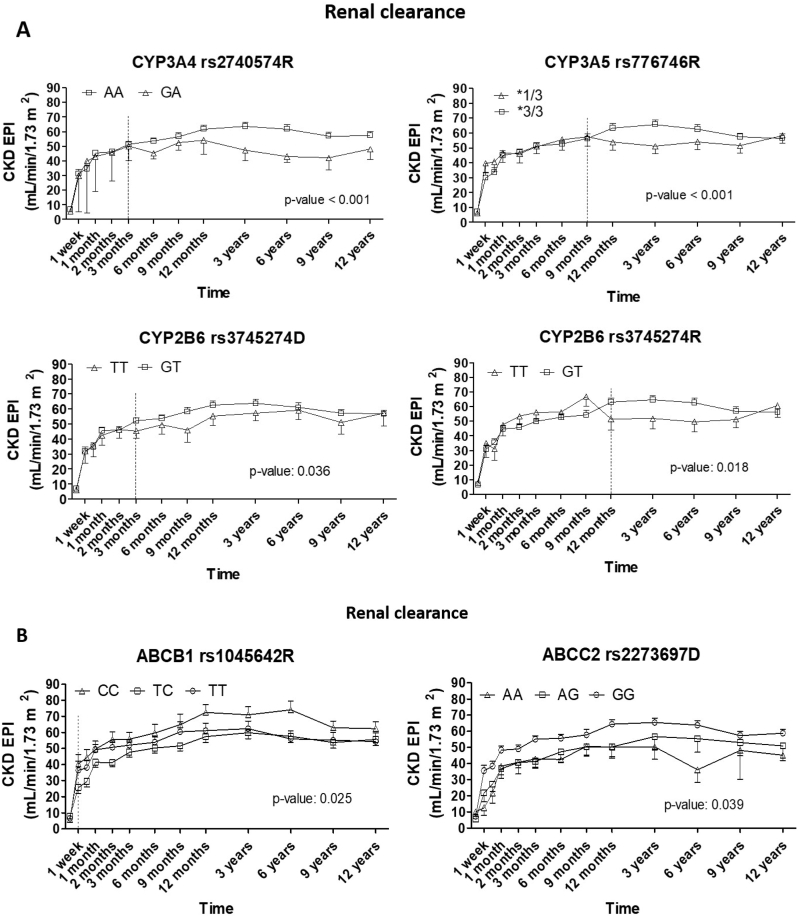
Renal clearance (CKD-EPI) expressed as ml/min/1.73 m^2^ during the follow-up grouped by pharmacogene variants. Statistical test: *t*-test when there were 2 only possible variants; one-way ANOVA for 3 or more. Dot lines represent the moment from which the difference appears, and the statistical test is performed.

Renal function, based on its clearance capacity, was calculated for each patient at each sampling time during the follow-up. A normal eGFR is greater than 90 ml/min/1.73 m^2^, but values as low as 60 ml/min/1.73 m^2^ are considered normal if there is no other evidence of kidney disease. Thus, the desirable goal is to achieve a minimum estimated glomerular filtration rate of 60 ml/min/1.73 m^2^. Patients were grouped based on their SNP variants in pharmacogenes associated with clinical parameters, and their CKD-EPI (Chronic Kidney Disease Epidemiology Collaboration) rate of clearance was compared. Statistically significant differences mediated by variants in metabolic (Fig. 4A) and transport (Fig. 4B) genes were represented as ml/min/1.73 m^2^. All patients presented very poor function at the time of transplantation, with a renal clearance rate below 10 ml/min/1.73 m^2^. This rate tended to normalize during the first year, reaching around 50–60 ml/min/1.73 m^2^, with different success and time of recovery depending on gene variants. The AA variant in CYP3A4 rs2745074 SNP of recipient mediated better recovery of renal clearance than GA (60 and 45 ml/min/1.73 m2, respectively; p-value <0.001), which was observable from 3 months post-transplantation and remained throughout the entire follow-up. Variants ∗3/∗3 in CYP3A5 rs776746 of donor and GT in CYP2B6 rs3745274 of recipient mediated a higher rate of renal clearance than ∗1/∗3 (p-value <0.001) and TT (p-value: 0.018), respectively. In this case, the differences between groups were not observed until 9 and 12 months post-transplantation, respectively. Those patients receiving a kidney graft from a donor carrying the TT variant in the same CYP2B6 SNP (rs3745274) presented delayed and lower recovery of renal function compared to those with the GT variant (p-value: 0.036).

When considering variants present in active transport genes, statistically significant differences were observed in the ABCB1 and ABCC2 genes. CC variants in rs1045642 showed early and more effective recovery, with renal clearance remaining higher than in TC and TT patients, with levels over 60 ml/min/1.73 m2. Patients transplanted with organs from donors with GG variants in ABCC2 rs2273697 had better renal clearance throughout the entire follow-up, with differences observed from the first week after transplantation onwards (p-value: 0.039), with sustained levels around 60 ml/min/1.73 m^2^.

### 3.7 Interactions of pharmacogene variants associated with clinical parameters

**Fig. 5:**
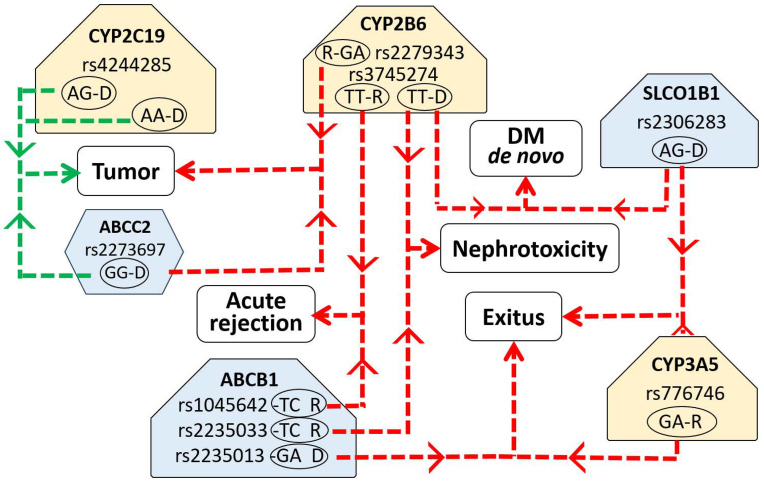
Scheme of significant associations between pharmacogenes variants interactions and the clinical parameters studied. In each gene variant, R: Recipient and D: Donor; DM: diabetes mellitus; Green lines: reduced risk; Red lines: increased risk; All lines present the same thickness because the OR is below 0.2 (green) o above 5 (red) in all cases; Yellow nodes: variants in metabolizer genes; Blue nodes: variants in transporter genes.

The effect of pharmacogenetics interactions on the risk of clinical events occurrence was also studied. The interaction between variants located in genes encoding proteins involved in drug transport and/or metabolism reported significant associations with different clinical outcomes (Fig. 5). The combination in donor of GG variant in ABCC2 rs2273697 with AG or AA variants in rs4244285 of CYP2C19 metabolizer gene could reduce the risk of tumor occurrence, with an OR < 0.2 in both cases. The combination of some variants in CYP2B6 metabolizer gene with different variants in transporter genes mediated significantly increased risk of clinical events incidence. In this sense, GA variant in rs2279343 of recipient when coinciding with GG variant in ABCC2 rs2273697 of donor could increase the risk of tumor occurrence with a 5.3 odds ratio. The TT variant in recipient rs3745274 of CYP2B6 was also associated with increased risk of graft acute rejection if combined with TC variant in ABCB1 rs1045642 of recipient. If TT gene variant was present in rs3745274 of CYP2B6 in donor, the risk of both nephrotoxicity and DM de novo could increase if concur with TC variant in ABCB1 rs2235033 of recipient or AG variant in SLCO1B1 rs2306283 of donor, respectively (OR: 7.5 and 9.8). The presence of GA variant (∗1/∗3) in CYP3A5 rs776746 of recipient could be associated with increased risk of exitus during the follow-up when coinciding with GA variant in ABCB1 rs2235013 of donor or AG variant in SLCO1B1 rs2306283 of donor (OR: 6.4 in both cases).

## 4 Discussion

In this study, we assessed the association between pharmacogene variants and clinical parameters using a panel of 37 SNPs from 14 genes—previously reported to influence drug management—in both donors and recipients within a cohort of 79 kidney transplant patients. We examined these associations in relation to the most frequent clinical parameters observed during a 12-year follow-up. Our findings indicate the presence of associations between pharmacogene variants and clinical outcomes, including survival, renal function, and tumor occurrence. Specifically, our results show that: a) 12-year post-transplant survival was associated with variants in both metabolism (CYP3A5 and CYP2B6) and transporter (ABCB1 and ABCC2) genes; b) increased risk of tumor occurrence associated with ABCC2 variants in donors and, conversely, reduced risk was observed in recipients with the GT variant in CYP2B6 rs3745274; c) better recovery of renal function was associated with variants in transporter (ABCB1 and ABCC2) and metabolism (CYP3A5, CYP3A4, and CYP2B6) genes; d) variants in CYP3A4, CYP3A5, and CYP2C19 metabolism genes, as well as ABCB1 and SLCO1B1 transporter genes influenced different tacrolimus pharmacokinetics during follow-up; e) other pharmacogene variants were associated with clinical parameters such as diabetes mellitus and infections.

The most commonly reported gene-drug interaction in organ transplantation involves the single nucleotide polymorphism (SNP) rs776746 A > G in CYP3A5 (which defines the ∗1 and ∗3 haplotypes) and tacrolimus (Brunet and Pastor-Anglada, 2022; van Gelder et al., 2020; Krall et al., 2021; Genvigir et al., 2020). This interaction has been assigned the highest level of scientific evidence (1A) for metabolism/pharmacokinetics and dosage by the PharmGKB database. The presence of the G variant at this location results in altered mRNA splicing, leading to a premature stop codon and the production of a non-functional CYP3A5 protein (Urzì Brancati et al., 2022). Patients with the ∗3/∗3 haplotype, known as non-expressers, exhibit increased tacrolimus concentrations due to impaired drug metabolism. The Clinical Pharmacogenetics Implementation Consortium (CPIC) and the Dutch Pharmacogenetics Working Group (DPWG) have issued dosing recommendations based on CYP3A5 haplotypes, advising a starting dose 1.5- to 2.5-fold higher than the standard dose for ∗1/∗3 (intermediate metabolizers) and ∗1/∗1 (extensive metabolizers) patients, respectively.

Additionally, the CYP3A4 ∗1B variant (rs2740574) has been associated with enhanced CYP3A4 activity, and carriers of this variant exhibit a lower tacrolimus dose-adjusted concentration ratio (C/D) compared to CYP3A4 ∗1/∗1 patients (Degraeve et al., 2020; Šimičević et al., 2022). Variants in other genes involved in the pharmacokinetics of drugs used in solid organ transplantation, such as ABCB1, ABCC2, CYP2C19, and UGT1A9, have also been associated with tacrolimus metabolism (Urzì Brancati et al., 2022), although their relationships with immunosuppressive drugs are less consistent. These genes may also play a role in the metabolism of other medications used in transplantation settings. Regarding ABCB1, it encodes an ATP-dependent efflux pump that mediates the excretion of tacrolimus and its metabolites into urine, promoting drug clearance (Degraeve et al., 2020). Decreased expression or function of this protein due to pharmacogene variants, such as rs1045642 and rs9282564, can affect tacrolimus pharmacokinetics and has been associated with an increased risk of nephrotoxicity (Tziastoudi et al., 2021; Hawwa et al., 2009), although discrepant results have been reported. Moreover, the risk of graft rejection has been linked to rs1045642 and rs2032582 SNPs in ABCB1 (Korkor et al., 2023). ABCC2 may also be involved in tacrolimus transport, as the rs3740066 SNP has been shown to significantly increase dose-normalized tacrolimus concentrations, whereas rs717620 had no significant effect (Li et al., 2011). However, long-term studies on these associations are lacking. Variants in UGT1A9 (rs72551330, rs3832043, rs6714486, and rs17868320) have been associated with mycophenolate mofetil toxicity and efficacy in kidney transplantation (Krall et al., 2021), although the clinical evidence remains low (levels 3 and 4). Associations between CYP2C19 (∗2, rs4244285) and increased hospital stays in kidney transplant patients treated with tacrolimus have been reported (evidence level 3) (Bosó et al., 2013), although this finding has not been confirmed. The CC variant in the NOD2 gene has also been associated with prolonged hospital stays (Bosó et al., 2013). To date, no associations between clinical events and variants in genes such as CYP2B6, SLCO1B1, and CYP2C9 have been reported. It is important to note that long-term studies on the impact of pharmacogenetic variants on the outcomes of transplant patients are still lacking.

Given the frequent comorbidities in transplanted patients, their survival rates are often reduced. We evaluated the association between the risk of exitus during follow-up (and its timing) and the gene variants included in our panel. Logistic regression analysis revealed that variants in four SNPs of ABCB1, CYP3A5, CYP2B6, and ABCC2 genes were associated with altered risk of death from any cause after 12 years. However, after applying false discovery rate (FDR) penalization, only variants in donors' ABCB1 and ABCC2 genes remained significantly associated with reduced (OR: 0.116) and increased (OR: 4.133) risk, respectively. While these variants have been previously linked with tacrolimus, none of the ABCB1 SNPs had robust scientific evidence of drug effects (levels of evidence 3 and 4 in PharmGKB©). Kaplan-Meier analysis of variants significantly associated with survival showed that only ABCB1 rs9282564 (log-rank: 0.042) and ABCC2 rs2273697 (log-rank: 0.005) in donors had significant differences in survival rates. To evaluate the effect of CYP3A5 rs776746, a frequently associated variant with tacrolimus, Kaplan-Meier analysis was performed on our cohort subdivided into ∗1/∗3 and ∗3/∗3 CYP3A5 haplotypes. We did not observe statistically significant differences (log-rank: 0.138) between haplotypes; however, CYP3A5 ∗1/∗3 recipients had reduced survival probability compared with ∗3/∗3 patients in the later stages of follow-up, suggesting a potential long-term effect on patient outcomes.

Regarding tumor occurrence, our results indicate that the GT variant in CYP2B6 rs3745274 in recipients was statistically associated with an increased risk of tumor incidence during follow-up (p-value: 0.048). Conversely, this variant was also associated with a reduced risk of diabetes mellitus occurrence (p-value: 0.056). However, we did not find strong evidence linking CYP2B6 variants with drug use in transplantation or short-term cancer risk studies.

Renal dysfunction is commonly reported in organ-transplanted patients, which can result from graft rejection or the effects of administered treatments, particularly tacrolimus, known to cause nephrotoxicity in some patients with elevated blood concentrations of drug (Knops et al., 2023; Sallustio et al., 2021). These elevated levels may arise from incorrect dosing or individual variations in drug metabolism and excretion. We assessed the potential association of pharmacogene variants with clinical parameters affecting renal function, including nephrotoxicity, graft rejection, and the need for re-transplantation. Our results indicate that the ABCB1 gene could influence the risk of chronic rejection (GA variant in donor's rs2235013 SNP; OR: 0.13) and severe infections, either reducing it (CC variant in recipient's ABCB1 rs1045642; OR: 0.087) or increasing it (AA variant in donor's ABCB1 rs9282564; OR: 23.8). Acute rejection was associated with variants in CYP3A4, CYP2B6, UGT1A9 metabolism genes (with CYP3A4 and CYP2B6 having protective roles and UGT1A9 increasing risk), and the SLCO1B1 transporter gene, where the TT variant in recipient rs4149056 showed a reduced risk of acute rejection (OR: 0.157). Regarding the need for re-transplantation, variants in TPMT and CYP2C9 genes were associated with a protective role, though we could not determine the underlying rationale in the literature.

The AA (TT) variant in CYP3A4 rs2740574 has been previously associated with increased tacrolimus concentration/dose (C/D) (Kuypers et al., 2007; Hannachi et al., 2021), which aligns with our findings of significantly higher (p-value <0.001) C/D in AA recipients. This may account for reduced immune activity and decreased rejection risk. CYP3A5 ∗3 haplotype has also been associated with increased dose-adjusted tacrolimus concentrations (Contreras-Castillo et al., 2021; Cheng et al., 2022), consistent with our observations. Recipients with the TT variant in SLCO1B1 rs4149056 showed higher dose-adjusted tacrolimus concentrations, possibly mediating a greater immunosuppressive effect and protecting against rejection, consistent with our logistic regression results (OR: 0.157). Although a previous study did not find significant effects on tacrolimus concentrations (Oetting et al., 2018), we observed significantly sustained lower dose-adjusted tacrolimus concentrations over time in patients receiving grafts from donors with the AAgenotype in this SNP, supporting the protective role against nephrotoxicity. Indeed, the AA variant in this SNP in both recipient and donor patients significantly reduced (Table 3) the risk of nephrotoxicity (OR: 0.076 and 0.108, respectively). On the other hand, the CC variant in NOD2 rs2066844 in donor also showed a reduced risk of nephrotoxicity, although no significant effect was observed on tacrolimus pharmacokinetics. A previous work (Bosó et al., 2013) reported a significant association of this variant with increased hospital stay. Our study also found a strong association (p-value: 0.026) with a substantial increase in nephrotoxicity risk (OR: 11.6) in patients with the AA variant in CYP2C19 rs4244285, which defines the ∗2 poor metabolizer haplotype and has been reported (Huang et al., 2022) to require lower tacrolimus doses for therapeutic concentrations. Our results confirm this, with significantly higher (p-value: 0.026) dose-adjusted tacrolimus concentrations (over 300 [(ng/ml)/(mg/kg)]) observed in patients receiving a kidney with this variant, which could contribute to nephrotoxicity. CYP2C19 metabolizes omeprazole, a proton pump inhibitor used by all patients in our cohort, and pharmacogenetics in this gene can alter its pharmacokinetics (Román et al., 2014). The ∗2 allele (A variant in rs4244285) reduces its metabolizing efficacy, and our group previously reported (Bosó et al., 2013) that patients with the AA genotype in CYP2C19 rs4244285 required prolonged hospital stays post-transplantation. This blocks omeprazole metabolism by CYP2C19, making it a substrate for CYP3A5, which competes with tacrolimus, leading to its inefficient metabolism and accumulation in the body. Thus, evaluating not only direct pharmacogene-drug associations but also drug-pharmacogene-drug and pharmacogene-pharmacogene-drug interactions is crucial. In this sense, the pharmacogenes interactions were also analyzed in this work since these could improve the predictive perspective of pharmacogenetics and relevant associations were found.

Gene variants that already proved direct association with exitus risk and with tacrolimus pharmacokinetics and renal clearance alteration (CYP3A5 rs776746) also showed interactions with variants in SLCO1B1 (rs2306283) and ABCB1 (rs2235013) that increased this risk of exitus. Other genes with direct association with tumor, exitus risk and renal clearance (CYP2B6 and ABCC2) also proved interactions with variants in other genes that significantly associated with increased risk of acute rejection, nephrotoxicity (both with ABCB1 variants, which were also proved to alter tacrolimus pharmacokinetics and renal clearance by themselves). The latter also occurred in DM de novo but with SLCO1B1 variants, which were associated with tacrolimus pharmacokinetics changes. These results support the idea that pharmacogenes interactions can play a significant role in the predictive perspective of pharmacogenetics studies. Genotyping patients with a comprehensive pharmacogenetics panels and following their long-term outcomes has highlighted associations with clinical events that may emerge later, as previously observed in our liver transplantation cohort (Sendra et al., 2022). While larger validation studies are needed to confirm these results, pharmacogenetic testing could provide valuable information for clinicians to improve pharmacotherapy management (Lloberas et al., 2023; Vidal-Alabró et al., 2024) and the clinicians’ predictive accuracy.

It must be taken into account that the long-term use of immunosuppressive drugs following kidney transplantation increases the risk of malignancies, life-threatening infections, nephrotoxicity, and, paradoxically, eventual allograft rejection. Emerging therapeutic strategies aim to achieve a balance between over- and under-immunosuppression by modulating immune responses through various approaches, including antibody-based therapies, as well as cell and gene therapies. Antibody-based therapies focus on blocking co-stimulatory immune activation signals. For instance, Belatacept, a fusion protein targeting CD80 and CD86, inhibits the entire co-stimulation pathway mediated by CD28 or CTLA4 (Noble et al., 2025). Alternatively, VEL-101, a monoclonal antibody targeting CD28, offers a more specific and controlled modulation of T cell activation (Khan et al., 2025). Genetic variants in these targets and associated pathways should be considered when selecting the most appropriate therapeutic regimen. In this context, polymorphisms in the IL10 gene—which encodes a key cytokine involved in immunomodulatory processes—have been associated with variable tumor susceptibility (Li et al., 2022). Understanding these genetic variants facilitates the development of personalized advanced therapies, including both cell- and gene-based strategies. In terms of cell therapy, the use of regulatory T cells (Tregs) (Matarneh et al., 2025), which play a central role in maintaining immune tolerance, and regulatory B cells (Bregs) (McNee et al., 2025), which contribute to allograft protection, has shown promising results in transplantation studies. Regarding gene therapy, our group proposed a model for the ex vivo transfer of the human IL10 gene to liver grafts in a porcine model (Sendra et al., 2024). This was achieved using non-viral hydrodynamic injection during back-table surgery, prior to implantation. The procedure was demonstrated to be safe, and the overall results support its potential clinical relevance.

In conclusion, our findings in this work suggest that variants in genes encoding proteins involved in drug metabolism and transportation may be associated with clinical parameters of high incidence in transplant recipients. Our results confirm that variants in CYP3A5 affect tacrolimus pharmacokinetics that could require dose adjustment, as widely reported. We also found significantly increased risk of exitus during the follow-up through a direct association, which was even enhanced through the interaction of CYP3A5 with specific variants in ABCB1 and SLCO1B1 transporter genes. Contrarily, although CYP2B6 has not been previously associated with transplantation clinical events, in our cohort, some variants were found to alter the renal clearance and increase the risk of exitus, tumor, acute rejection and DM occurrence during the 12-year follow-up, which were enhanced if coincided with variants in ABCC2, SLCO1B1 and ABCC2 transporter genes. Thus, identifying gene multiple variants with panels would favor the predictive potential of pharmacogenetics and help clinicians to optimize personalized pharmacotherapy.

## 5 Study limitations

This study presents data from a very long-term follow-up of kidney transplant recipients, which has been done only rarely. However, the cohort size is limited, and all patients were transplanted and followed at a single center. Therefore, further multicenter studies with larger cohorts and greater genetic diversity are needed to validate the findings reported in this paper.

## CRediT authorship contribution statement

**Luis Sendra:** Software, Methodology, Investigation, Writing – original draft. **Gladys G. Olivera-Pasquini:** Data curation, Investigation. **Enrique G. Zucchet:** Investigation. **Fabiana D.V. Genvigir:** Data curation. **María Isabel Beneyto:** Visualization. **Julio Hernández-Jaras:** Methodology. **María José Herrero:** Conceptualization, Validation, Supervision. **Salvador F. Aliño:** Conceptualization, Writing – review & editing, Supervision.

## Declaration of competing interest

The authors have nothing to declare.

## Data Availability

The authors do not have permission to share data.
