# Supplementary material for: Pharmacogenetics association with long-term clinical evolution in a kidney transplant patients cohort
Source: Curr Res Pharmacol Drug Discov. 2025 Jul 24;9:100230. doi: 10.1016/j.crphar.2025.100230 (PMC12319246; doi:10.1016/j.crphar.2025.100230)
Supplement: Multimedia component 1 [file mmc1.docx]

**Supplementary** **material**

Table S1. SNPs included within the pharmacogenetics panel.

| **Gene** | **SNP** | |
| --- | --- | --- |
| *abcb1* | rs1045642 | rs2235013 |
|  | rs1128503 | rs2235033 |
|  | rs2032582 | rs3213619 |
|  | rs229109 | rs9282564 |
| *abcc2* | rs3740066 | rs717620 |
|  | rs2273697 |  |
| *abcg2* | rs2231137 | rs2231142 |
| *cyp2b6* | rs2279343 | rs3745274 |
| *cyp2c19* | rs4244285 |  |
| *cyp2c9* | rs1799853 | rs1057910 |
| *cyp3a4* | rs2740574 |  |
| *cyp3a5* | rs41303343 | rs776746 |
|  | rs10264272 |  |
| *mthfr* | rs1801131 | rs1801133 |
| *nod2* | rs2066844 | rs2066845 |
| *slco1a2* | rs11568564 | rs72559749 |
|  | rs11568563 |  |
| *slco1b1* | rs2306283 | rs4149056 |
| *tpmt* | rs1142345 | rs1800462 |
|  | rs1800460 |  |
| *ugt1a9* | rs17868320 | rs72551330 |
|  | rs6714486 |  |

Table S2. Distribution of patients based on their variants in pharmacogene variants associated with exitus probability and the occurrence of exitus.

| **Exitus** |  |  |  |  |  |  |  |  |
| --- | --- | --- | --- | --- | --- | --- | --- | --- |
|  |  |  |  |  | **Clinical event** | | | |
| **Gene** | **SNP** | **D/R** | **Variant** | **Patients** | **Absence** | | **Presence** | |
|  |  |  |  | n | n | % | n | % |
| ABCB1 | rs9282564 | D | AA | 73 | 51 | 69.9 | 22 | 30.1 |
|  |  |  | AG | 6 | 2 | 33.3 | 4 | 66.7 |
| ABCC2 | rs2273697 | D | AA | 4 | 1 | 25 | 3 | 75 |
|  |  |  | GA | 16 | 7 | 43.8 | 9 | 56.2 |
|  |  |  | GG | 59 | 45 | 76.3 | 14 | 23.7 |
| CYP2B6 | rs3745274 | R | GT | 68 | 48 | 70.6 | 20 | 29.4 |
|  |  |  | TT | 11 | 5 | 45.5 | 6 | 54.5 |
| CYP3A5 | rs776746 | R | GA | 16 | 8 | 50 | 8 | 50 |
|  |  |  | GG | 63 | 45 | 71.4 | 18 | 28.6 |

SNP: Single Nucleotide Polymorphism; D: donor; R: recipient;

Table S3. Distribution of patients based on their variants in pharmacogene variants associated with the probability of tumor occurrence.

| **Tumor** |  |  |  |  |  |  |  |  |
| --- | --- | --- | --- | --- | --- | --- | --- | --- |
|  |  |  |  |  | **Clinical event** | | | |
| **Gene** | **SNP** | **D/R** | **Variant** | **Patients** | **Absence** | | **Presence** | |
|  |  |  |  | n | n | % | n | % |
| CYP2B6 | rs3745274 | R | GT | 68 | 51 | 75 | 17 | 25 |
|  |  |  | TT | 11 | 7 | 63.6 | 4 | 36.4 |
| ABCC2 | rs2273697 | D | AA | 4 | 1 | 25 | 3 | 75 |
|  |  |  | GA | 16 | 9 | 56.3 | 7 | 43.8 |
|  |  |  | GG | 59 | 48 | 81.4 | 11 | 18.6 |
| ABCB1 | rs9282564 | D | AA | 73 | 55 | 75.3 | 18 | 24.7 |
|  |  |  | AG | 6 | 3 | 50 | 3 | 50 |

SNP: Single Nucleotide Polymorphism; D: donor; R: recipient;

Table S4. Distribution of patients based on their variants in pharmacogene variants associated with the probability of occurrence of clinical events involved in kidney function.

| **Clinical parameter** |  |  |  |  |  | **Clinical event** | | | |
| --- | --- | --- | --- | --- | --- | --- | --- | --- | --- |
|  | **Gene** | **SNP** | **D/R** | **Variant** | **Patients** | **Absence** | | **Presence** | |
|  |  |  |  |  | n | n | % | n | % |
| **Chronic rejection** | ABCB1 | rs2235013 | D | AA | 28 | 23 | 82.1 | 5 | 17.9 |
|  |  |  |  | GA | 40 | 36 | 90 | 4 | 10 |
|  |  |  |  | GG | 11 | 6 | 54.5 | 5 | 45.5 |
| **Acute rejection** | CYP3A4 | rs2740574 | R | AA | 75 | 58 | 77.3 | 17 | 22.7 |
|  |  |  |  | GA | 4 | 1 | 25 | 3 | 75 |
|  | SLCO1B1 | rs4149056 | R | CC | 3 | 2 | 66.7 | 1 | 33.3 |
|  |  |  |  | TC | 20 | 12 | 60 | 8 | 40 |
|  |  |  |  | TT | 56 | 45 | 80.4 | 11 | 19.6 |
|  | CYP2B6 | rs3745274 | D | GT | 67 | 53 | 79.1 | 14 | 20.9 |
|  |  |  |  | TT | 12 | 6 | 50 | 6 | 50 |
|  | UGT1A9 | rs6714486 | D | TA | 7 | 3 | 42.9 | 4 | 57.1 |
|  |  |  |  | TT | 71 | 56 | 78.9 | 15 | 21.1 |
| **Nephrotoxicity** | SLCO1B1 | rs2306283 | R | AA | 25 | 22 | 88 | 3 | 12 |
|  |  |  |  | AG | 43 | 33 | 76.7 | 10 | 23.3 |
|  |  |  |  | GG | 11 | 5 | 45.5 | 6 | 54.5 |
|  | SLCO1B1 | rs2306283 | D | AA | 21 | 16 | 76.2 | 5 | 23.8 |
|  |  |  |  | AG | 45 | 38 | 84.4 | 7 | 15.6 |
|  |  |  |  | GG | 13 | 6 | 46.2 | 7 | 53.8 |
|  | CYP2C19 | rs4244285 | D | AA | 5 | 2 | 40 | 3 | 60 |
|  |  |  |  | GA | 18 | 13 | 72.2 | 5 | 27.8 |
|  |  |  |  | GG | 56 | 45 | 80.4 | 11 | 19.6 |
|  | NOD2 | rs2066844 | D | CC | 71 | 53 | 74.6 | 18 | 25.4 |
|  |  |  |  | CT | 6 | 6 | 100 | 0 | 0 |
|  |  |  |  | TT | 2 | 1 | 50 | 1 | 50 |
|  | ABCG2 | rs2231137 | R | GA | 4 | 1 | 25 | 3 | 75 |
|  |  |  |  | GG | 75 | 59 | 78.7 | 16 | 21.3 |
| **Re-transplantation** | TPMT | rs1142345 | D | AA | 74 | 68 | 91.9 | 6 | 8.1 |
|  |  |  |  | GA | 5 | 3 | 60 | 2 | 40 |
|  | CYP2C9 | rs1799853 | D | CC | 65 | 60 | 92.3 | 5 | 7.7 |
|  |  |  |  | CT | 14 | 11 | 78.6 | 3 | 21.4 |

SNP: Single Nucleotide Polymorphism; D: donor; R: recipient;

Table S5. Distribution of patients based on their variants in pharmacogene variants associated with the occurrence probability of DM de novo and infections.

| **Clinical parameter** |  |  |  |  |  | **Clinical event** | | | |
| --- | --- | --- | --- | --- | --- | --- | --- | --- | --- |
|  | **Gene** | **SNP** | **D/R** | **Variant** | **Patients** | **Absence** | | **Presence** | |
|  |  |  |  |  | n | n | % | n | % |
| **DM de novo** | CYP2B6 | rs3745274 | R | GT | 68 | 46 | 67.6 | 22 | 32.4 |
|  |  |  |  | TT | 11 | 4 | 36.4 | 7 | 63.6 |
| **Infections** | ABCB1 | rs1045642 | R | CC | 20 | 19 | 95 | 1 | 5 |
|  |  |  |  | TC | 39 | 24 | 61.5 | 15 | 38.5 |
|  |  |  |  | TT | 20 | 15 | 75 | 5 | 25 |
|  | ABCB1 | rs9282564 | D | AA | 73 | 11 | 15.1 | 62 | 84.9 |
|  |  |  |  | AG | 6 | 4 | 66.7 | 2 | 33.3 |

SNP: Single Nucleotide Polymorphism; D: donor; R: recipient;

Table S6. Genetic parameters associated with tacrolimus pharmacokinetics and renal clearance rate

| **GENETIC PARAMETERS** | | | | **CLINICAL PARAMETERS** | | | | |
| --- | --- | --- | --- | --- | --- | --- | --- | --- |
| **GENE** | **SNP** | **D/R** | **Variant** | **Tacrolimus Pharmacokinetics (C/D)** | | **Renal Clearance (CKD)** | | **Associated adverse event** |
|  |  |  |  | Significance | Change | Significance | Change |  |
| **ABCB1** | rs2235013 | D | AA | **🗸** | **↑** | **🗸** | **↓** | Chronic rejection |
|  | rs2235033 | R | TT | **🗸** | **↑** | **🗸** | **↓** | Tumor |
|  | rs1045642 | R | TT | **🗸** | **↑** | **🗸** | **↓** | Infection |
| **CYP3A4** | rs2740574 | R | AA | **🗸** | **↑** | **🗸** | **↑** | Acute rejection |
| **CYP3A5** | rs776746 | R | *3/*3 | **🗸** | **↑** | **🗸** | **↑** | Exitus |
| **CYP2C19** | rs4244285 | D | AA | **🗸** | **↑** | Ø | − | Nephrotoxicity |
| **SLCO1B1** | rs2306283 | D | GG | **🗸** | **↑** | Ø | − | Nephrotoxicity |
|  | rs4149056 | R | TT | **🗸** | **↑** | Ø | − | Acute rejection |
| **ABCC2** | rs2273697 | D | GG | Ø | − | **🗸** | **↑** | Exitus |
|  |  |  |  |  |  |  |  | Tumor |
| **CYP2B6** | rs3745274 | D | GT | Ø | − | **🗸** | **↑** | Acute rejection |
|  |  | R | GT | Ø | − | **🗸** | **↑** | Exitus |
|  |  |  |  |  |  |  |  | Tumor |
|  |  |  |  |  |  |  |  | Diabetes mellitus |
